# Molecular Modeling of Indeno [1, 2-b] Quinoline-9, 11-Diones as Cytotoxic Agents

**Published:** 2018

**Authors:** Ramin Miri, Fatemeh Bohlooli, Nima Razzaghi-Asl, Ahmad Ebadi

**Affiliations:** a *Medicinal and Natural Products Chemistry Research Center, Shiraz University of Medical Sciences, Shiraz, Iran.*; b *Department of Medicinal Chemistry, School of Pharmacy, Ardabil University of Medical Sciences, Ardabil, Iran. *; c *Drug and Advanced Sciences Research Center, School of Pharmacy, Ardabil University of Medical Sciences, Ardabil, Iran. *; d *Department of Medicinal Chemistry, School of Pharmacy, Hamadan University of Medical Sciences, Hamadan, Iran. *; e *Medicinal Plants and Natural Products Research Center, Hamadan University of Medical Sciences, Hamadan, Iran.*

**Keywords:** Cancer, DNA, Indeno[1, 2-b]quinoline-9, 11-dione, Molecular modeling

## Abstract

Deoxyribonucleic acid (DNA) is an important molecular target for anti-cancer agents due to its involvement in gene expression and protein synthesis which are fundamental steps in cell division and growth. A number of antineoplastic agents interfere with DNA and hence disturb the cell cycle. Compounds including planar aromatic rings are privileged scaffolds in binding to DNA. This characteristic is mainly arisen from the fact that such structural feature may be appropriate to insert between the base pairs of the DNA double helix and produce relatively stable non-covalent complexes. Besides π-π stacking interactions, binding to the DNA molecule might be intensified through H-bond interactions of heterocyclic rings. In the present contribution, a series of experimentally validated cytotoxic indeno[1,2-b]quinoline-9,11-diones (1-12) and their aromatized analogues (13-21) developed in our group were subjected to docking and molecular dynamics simulations to elucidate their most probable binding modes with DNA.

## Introduction

Malignant tumors are responsible for cancer (1) and can be broken away and spread out to other tissues of the body (2). In spite of recent advances in treatment strategies, cancer is still one of the major causes of death worldwide (3). Many anti-cancer agents interact directly with DNA or prevent its processing through interaction with a definite enzyme (4, 5). Regarding to this, development of DNA targeting anti-cancer drugs is one of the considerable interests within pharmaceutical scientists.

Generally, ligand-DNA interactions can be categorized into two major classes; intercalation and groove binding (6). In intercalation mechanism, a planar aromatic/polyaromatic ligand with sufficiently large surface area inserts into the space between the base pairs of DNA double helix. The driving forces for intercalation are primarily π-π stacking interactions, H-bonds and ionic interactions between ligand and base pairs (7). It has been postulated that intercalation reduces the DNA helical twist and elongates the DNA, hence disturbing its normal function (8). 

Minor groove-binding agents usually fit to the helical turn of the DNA grooves via Van der Waals or hydrogen bond interactions (5). This structural complementary may be attributed to the torsional freedom of interfering ligands that provide compatibility with a twisted helical turn of the DNA grooves. Such molecular pattern may be envisaged as several unfused aromatic rings being connected through rotatable bonds (9). Major groove-binders have been less reported due to the requirement of larger molecular sizes in order to be fitted into the DNA. Major groove binding pattern have frequently been observed in peptides and proteins (10). 

X-ray crystallographic structures of DNA-ligand complexes (*http://www.rcsb.org*) provided studies on the molecular mechanism and structure binding relationship of DNA interfering agents. Such structural data facilitated the performance of target based hit/lead design strategies toward developing new DNA binding anti-neoplastic agents. 

In our previous work, a series of new imidazole substituted indeno [1,2-b] quinoline-9,11-dione derivatives were synthesized and evaluated for their cell-based cytotoxic effects on HeLa, LS180, Jurkat and MCF-7 human cancer cell lines using MTT assay (11). Prepared compounds exhibited no (IC_16_ >100 µM) to good (IC_16 _= 0.7 µM in Jurkat cell lines) anti-tumoral activities within evaluated cell lines. Due to the uncertainty of results in IC_50_ scale, IC_16_ values were used for our modeling studies. Prepared compounds had rigid aromatic/heteroaromatic rings constructed around a dihydropyridine (DHP) or pyridine core, an appropriate pattern for DNA intercalation and H-bonding. In the present contribution, docking and molecular dynamics methods were applied to elucidate the most probable DNA binding mode of cytotoxic indeno[1,2-b]quinoline-9,11-dione derivatives.

## Experimental

*Preparation of oligonucleotide structures*

Crystal structures of two DNA-ligand complexes with PDB IDs 1D32 (12) and 102D (13) were retrieved from the Protein Data Bank as representatives of macromolecular templates (14). For preparation of required DNA targets, co-crystallographic ligands DIT and TNT were respectively removed from the PDB files 1D32 and 102D and all the crystallographic water molecules were also eliminated from each PDB file. Missing hydrogens were added, non-polar hydrogens were merged to their attached carbon atoms and kollman charges were dedicated to the oligonucleotide receptors. All the pre-processing steps for receptor files were performed via AutoDock Tools program (ADT) (15, 16). In addition; all the oligonucleotide structures were optimized to minimize the crystallographic induced clashes via steepest descent method by Gromacs package (17).

*Preparation of ligand structures*

Twenty-one DHP derivatives were categorized into indeno [1, 2-b]quinoline-9,11-diones and their aromatized derivatives. 2D chemical structures were rendered by Marvin Sketch online chemical editor (18) and relevant SMILES strings were used to generate the 3D structures by free online 3D conformation generator Frog 1.0 software (19). 

*Molecular docking*

Advanced docking package AutoDock4.2 was used to dock the screened molecules. For this purpose, 3D optimized structures of indeno [1, 2-b]quinoline-9,11-diones and their aromatized derivatives in PDB format were used as input files for the AutoDock Tools (ADT) (15, 16). All the pre-processing steps for ligand structures were performed according to the previous procedure (20). Prepared molecules were docked into the 3D structure of oligonucleotides extracted from the crystallographic files (1D32 and 102D). 

Modeling of the DNA-ligand interactions were conducted within desired number of genetic algorithm runs (50 GA runs) and energy evaluations (10^6 ^and 5×10^5 ^for 1D32 and 102D, respectively) on the basis of docking validation study. Other AutoDock operating parameters were set at their default values. A map was assigned to each atom type of the ligand and DNA. Relevant maps were estimated using AutoGrid module (part of the AutoDock package). A size of the grid was set to the 50×50×50 Ǻ^3^ (distributed in the x, y, and z directions) and centered on the macromolecule with a spacing of 0.375 Ǻ to include the space occupied by the DNA double helix.

LIGPLOT program was used to generate the 2D binding interaction maps between DHP structures and DNA double helix (21).

*Molecular dynamics*

All MD simulations were performed using GROMACS v.4.6.5 (22) applying Amber99SB (23) force field. The compatible topologies of the ligands were prepared using the General Amber Force Field (GAFF) (24) and ANTECHAMBER suite of programs (25, 26), applying the semi-empirical AM1BCC charges (27, 28). DNA-ligand complexes were prepared based on the best docking pose associated with largest binding energy. 

DNA-ligand complex was centered in a dodecahedron box filled with TIP3P water (29). Enough number of Na^+^ atoms was added to achieve electro-neutrality. A distance of 1 nm was set between the box walls and each edge of complex. For all of the molecular dynamic simulations, the Leonard-Jones and electrostatic interactions was computed using a cutoff value of 1.2 nm. The leapfrog algorithm (30) using a 1fs time-step was used as an integrator for the equation of motion. All covalent bonds were constrained using LINCS algorithm (31).

Actual simulation was set up by minimizing the systems to make sure that introduced steric clashes during the preparing process were removed. During minimization, maximum number of 5000 steps was applied using steepest descent algorithm. After initial relaxation, the system was equilibrated for 200 ps using the NVT and NPT ensemble respectively. In NVT ensemble (constant number, volume, and temperature) temperature was set at 300 K using a velocity-rescale thermostat (modified Berendsen thermostat) (32) with a coupling constant of 0.1 ps. After temperature stabled at 300 K, NPT ensemble (constant number, pressure, and temperature) was performed with an isotropic pressure of 1 bar and a coupling constant of 2 ps by using a Parrinello-Rahman barostat (33). 

Position restraints were applied to the ligands and DNA during the NVT and NPT ensemble. Long range electrostatic interactions were computed utilizing the particle mesh Ewald method (34). Equilibration was followed by a 50 ns production run using the NPT ensemble with the velocity-rescale thermostat (32) and the Parrinello–Rahman barostat (33). Evaluation of MD simulations was performed by extracting data from the trajectory files produced during the simulations.

## Results and Discussion

*Internal validation*

The internal validation step was performed via extracting the structures of co-crystallized ligands ditercalinium (DIT) and propamidine (TNT) and re-docking them into the original crystallographic files of the d(CGCAAATTTGCG)_2_ and d (CGCG)_2_ oligonucleotides. Validation of the AutoDock method for each crystallographic structure was interpreted in terms of root mean square deviation (RMSD) of the Cartesian coordinates of the atoms of the ligand in the docked and crystallographic poses (RMSD < 2Ǻ). It should be noted that top-ranked AutoDock clusters in the output files were supported by high conformational population. Obtained results indicated that the parameters set for AutoDock were reliable for reproducing X-ray complex structures.

**Figure 1:**
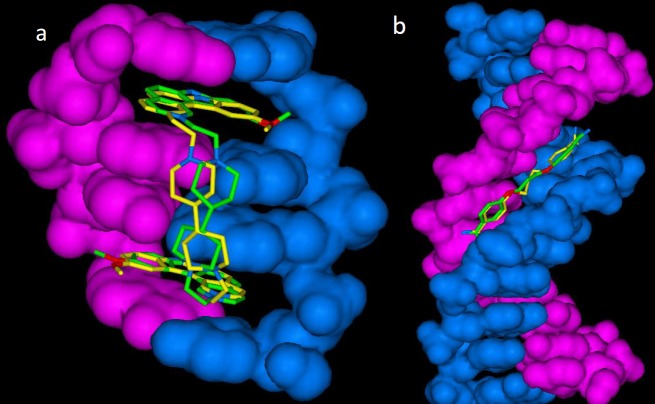
Lowest energy (bioactive conformation) poses of a**)** propamidine-DNA (1D32) and b**)** ditercalinium-DNA (102D) in docked (yellow stick) and crystallographic (green stick) complexes (DNA structure rendered in blue and pink as solvent-excluded surface) (RMSD for 1D32: 1.10 Å and RMSD for 102D: 1.05 Å)

For more elucidation, lowest energy poses of cognate drugs (propamidine and ditercalinium) in docked and crystallographic states are shown in Figure 1.

*Binding model selection*

AutoDock is a widely used docking package in computational medicinal chemistry and it is believed to offer relatively logical outcomes within several calculations (35). To further elucidate the interactions of DNA with indeno [1,2-b]quinoline-9,11-diones (1–21), we used AutoDock4.2 software to dock all the relevant structures into the d (CGCG)_2_ and d (CGCAAATTTGCG)_2 _oligonucleotides of DNA double helix. 

**Scheme 1:**
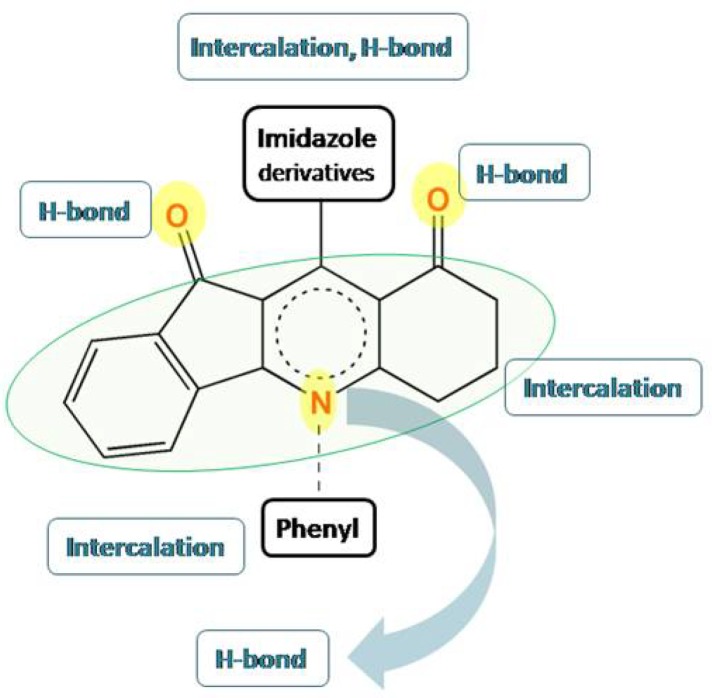
Possible relationship between chemical structure and DNA binding ability of evaluated indeno [1, 2-b] quinoline-9, 11-diones, as can be predicted from the model, both hydrophobic and hydrogen interactions might be expected on the basis of represented scaffold template

Structure elucidation of the evaluated indeno [1, 2-b]quinoline-9,11-diones showed that these compounds possessed aromatic/heteroaromatic rings constructed around a DHP/pyridine core. In our opinion, such molecular pattern might be appropriate for intercalation and H-bond interactions with DNA double helix. To explain more, possible structure DNA binding relationship of indeno [1, 2-b] quinoline-9,11-diones is depicted below (Scheme 1). Observed binding potentialities persuaded us toward performing modeling studies with the aim of proposing novel DNA interfering agents.

**Figure 2:**
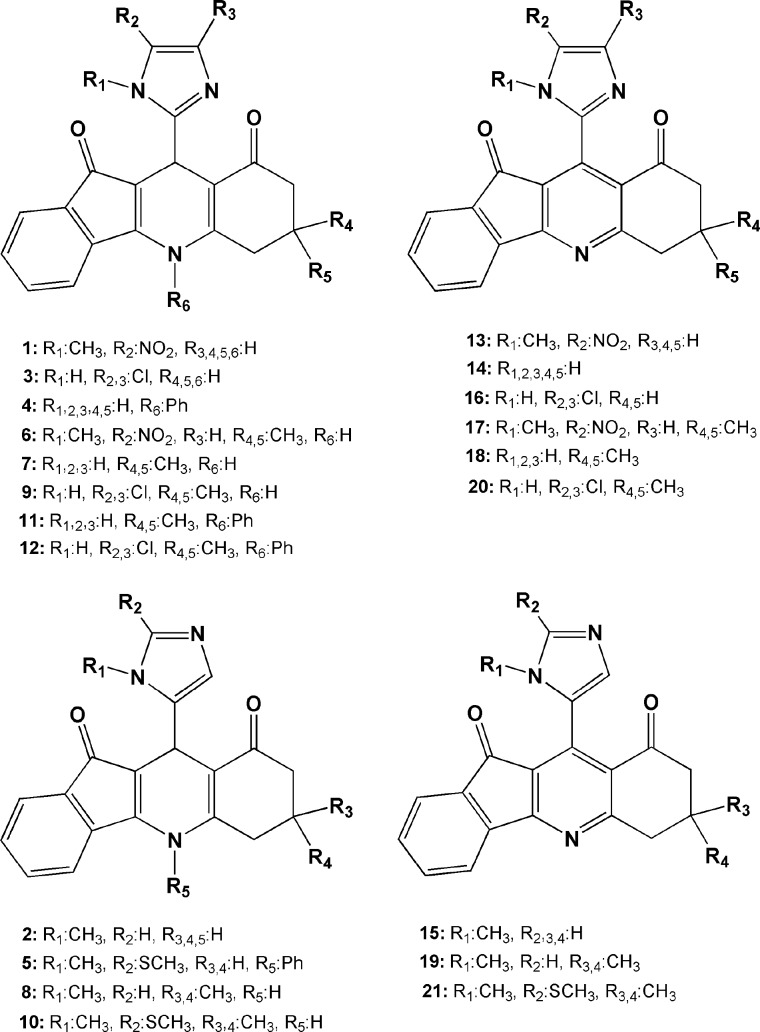
Chemical Structures of indeno [1, 2-b] quinoline-9,11-diones under study

Chemical structures of screened ligands are summarized in Figure 2. All of the molecules could be interpreted as drug-like chemical entities and for this reason they might be good candidates for further drug development strategies (36). In addition, selected DHP structures possessed low number of active torsions (rotatable bonds) which proposed them as suitable candidates for docking simulation due to lower free torsional energies.

For more clarification, *in-vitro* cytotoxicity effects of indeno [1, 2-b] quinoline-9,11-diones on different cell lines could be reviewed elsewhere (11). 

*Binding affinity prediction*

Predicted docked energies can be reported in terms of final docked energy (FDE) which is the sum of final intermolecular energy and final internal energy of ligand or estimated free energy change of binding (ΔG) that is the sum of final intermolecular energy and torsional free energy penalty (37). 

Docking results indicated that indeno [1, 2-b]quinoline-9,11-diones could be reversibly attached to DNA oligonucleotides with varied binding affinities ranging from -6.86 to -9.20 kcal.mol^-1^ and -5.85 to -9.87 kcal.mol^-1 ^in 1D32 and 102D macromolecular templates, respectively. For more clarification, AutoDock binding affinities together with probability factors in terms of number of clusters and top-ranked cluster population in AutoDock output files are summarized in Table 1.

**Table 1:** AutoDock results for the complex of indeno[1,2-b]quinoline-9,11-diones and DNA target

**No. of conformations in top-ranked cluster (out of 50)**	**No. of** **clusters**	**Inhibition constant (K**_i_ **)** **(µM)**	**Final Docked energy** **(kcal.mol** ^-1^ **)**	**Estimated free binding energy** **(kcal.mol** ^-1^ **)**	**PDB code**		**Comp. code**
41	4	5.58	-8.30	-7.17	1D32		**1**
30	3	4.60	-7.99	-7.28	1D32		**2**
29	3	4.68	-8.27	-7.27	1D32		**3**
46	3	2.36	-9.25	-7.68	1D32		**4**
24	4	4.86	-9.40	-7.25	1D32		**5**
45	4	5.85	-8.38	-7.14	1D32		**6**
32	4	3.98	-8.15	-7.37	1D32		**7**
38	3	5.64	-7.93	-7.16	1D32		**8**
44	2	2.13	-8.64	-7.74	1D32		**9**
43	4	4.28	-8.53	-7.32	1D32		**10**
49	2	2.16	-9.35	-7.73	1D32		**11**
50	1	1.08	-9.95	-8.14	1D32		**12**
50	1	2.53	-8.93	-7.63	1D32		**13**
27	3	0.764	-9.00	-8.34	1D32		**14**
45	2	2.02	-8.58	-7.77	1D32		**15**
2	8	0.179	-9.71	-9.20	1D32		**16**
37	5	9.34	-7.88	-6.86	1D32		**17**
21	5	0.624	-9.08	-8.45	1D32		**18**
34	4	4.39	-8.02	-7.31	1D32		**19**
4	6	0.270	-9.16	-9.12	1D32		**20**
29	5	5.47	-8.24	-7.18	1D32		**21**
48	2	6.65	-8.19	-7.06	102D		**1**
38	5	1.07	-8.76	-8.15	102D		**2**
34	2	0.134	-10.06	-9.38	102D		**3**
17	10	1.12	-9.18	-8.12	102D		**4**
14	6	4.80	-9.39	-7.26	102D		**5**
20	3	9.01	-8.13	-6.88	102D		**6**
31	4	0.480	-9.27	-8.62	102D		**7**
26	3	1.28	-8.82	-8.04	102D		**8**
36	4	0.328	-10.58	-8.85	102D		**9**
28	3	0.791	-9.44	-8.32	102D		**10**
24	5	1.64	-9.13	-7.89	102D		**11**
26	3	0.975	-9.42	-8.20	102D		**12**
48	2	1.73	-9.44	-7.86		102D	**13**
50	1	0.076	-10.32	-9.71		102D	**14**
26	2	0.304	-9.61	-8.89		102D	**15**
44	3	0.077	-10.44	-9.70		102D	**16**
21	7	3.94	-8.64	-7.37		102D	**17**
29	4	0.058	-10.5	-9.87		102D	**18**
35	3	0.340	-9.68	-8.82		102D	**19**
20	9	0.108	-10.18	-9.50		102D	**20**
18	7	0.844	-9.59	-8.29		102D	**21**

**Figure 3:**
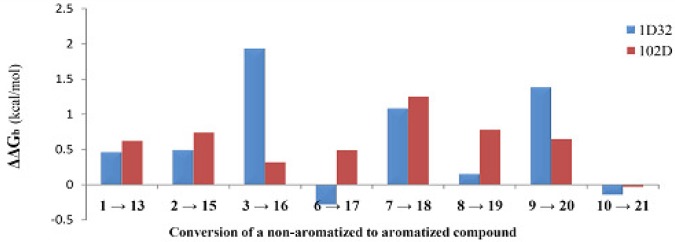
Comparison of the DNA binding affinities for non-aromatized (1,2,3,6,7,8,9 & 10) and aromatized (13,15,16,17,18,19,20 & 21) indeno [1,2-b] quinoline-9,11-diones (PDB deposition codes: 1D32 & 102D); as can be understood from the diagram, aromatized indeno [1, 2-b] quinoline-9,11-diones were associated with higher binding energies when compared to their non-aromatized analogues

**Figure 4:**
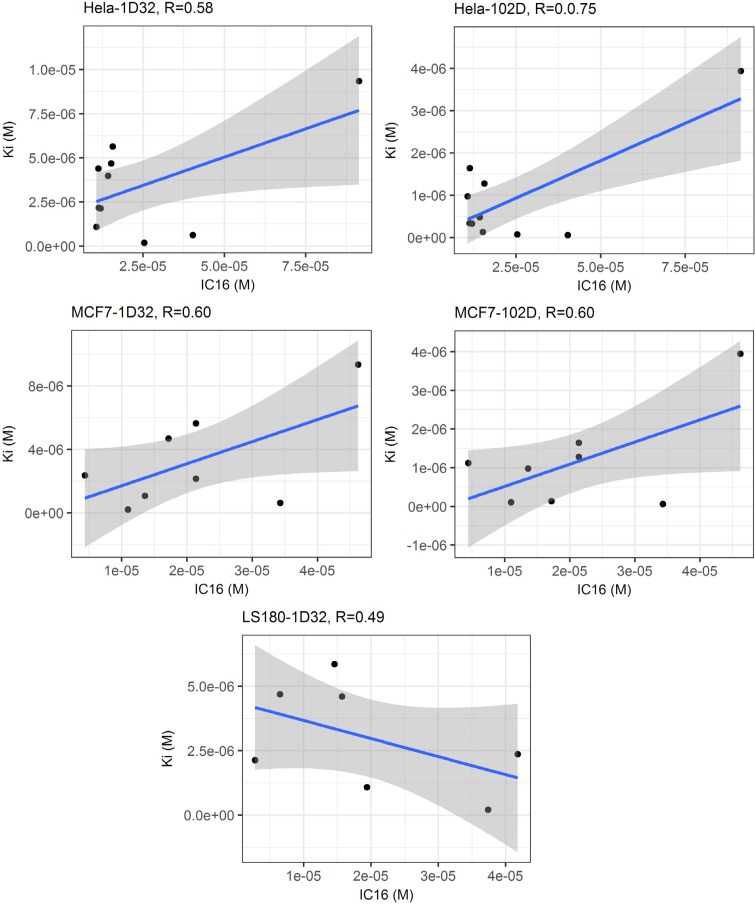
Linear regression analysis of predicted DNA binding affinities (Ki) versus experimental cytotoxic effects (IC_16_) within two DNA conformations (1D32 & 102D); it can be seen that maximum correlation was achieved in the case of HeLa cells (r = 0.75; r ^2^= 0.56 for experimental IC_16_ versus predicted K_i_ values in 102D system) while no correlation could be found in the case of Jurkat

Comparative analysis of experimental cytotoxicity values and calculated binding affinities might be interpreted via following rationales:

(1) Nearly all aromatized indeno [1, 2-b] quinoline-9,11-diones were associated with higher binding energies when compared to their non-aromatized analogues (Figure 3). One of the possible explanations for such observation might be attributed to the superior π-π stacking interactions with oligonucleotides of DNA double helix in aromatized compounds. Such observation could not be confirmed by experimental cytotoxic activities in Jurkat cell lines (Such rationalization may not be offered for other cell lines due to the uncertainty of cytotoxic data). This trend might be explained by this point that the cytotoxic effect of aromatized scaffolds in Jurkat cell lines is not related to the DNA intercalation.

(2) Experimental cytotoxic data showed that *N*-phenyl substituted compounds had increased effects in the majority of cases (Compounds 4, 5, 11 & 12). It was also recognized that FDE values (1D32) could better anticipate DNA-ligand interactions in such molecules (4: -9.25, 5: -9.40, 11: -9.35 & 12: -9.95 kcal/mol).

(3) FDE values over -10 kcal/mol were contributed to compounds 3, 9, 14, 16 & 20 in 102D model. Experimental results showed that except for compound 14 which was inactive in HeLa, LS180 and MCF7 cell lines, the other four compounds showed good anti-tumoral effects within tested cells. In relation to this, modeling studies confirmed that the incorporation of electron-withdrawing substituents such as chlorine atoms to the imidazole moiety of indeno [1, 2-b] quinoline-9, 11-dions might be associated with higher cytotoxic effects.

(4) Linear regression analysis of calculated binding affinities and experimental cytotoxicity values showed that maximum correlation could be achieved in the case of HeLa cells (r = 0.74; r^2 ^= 0.56 for experimental IC_16_ versus predicted K_i_ values in 102D system) (Figure 4). No correlation could be found between predicted DNA affinities and cytotoxic activities in Jurkat cell lines.

*Ligand-DNA binding interactions*

With the aim of further elucidating ligand-DNA binding interactions, LIGPLOT program was used to generate the binding interactions between DHPs and DNA. Selective hydrophobic interactions were depicted using the hydrophobic bond module of Ligplot (Supplementary 

material 1).

To be informative, binding interactions along with binding participants of various complexes of indeno [1, 2-b] quinoline-9,11-dions with DNA are summarized in the supplementary material 2. Analysis of the DNA-ligand binding interactions revealed that:

1) Various PDB deposited templates of DNA cold have a considerable effect on binding mode of indeno [1 ,2-b]quinoline-9,11-diones. Such observation might emphasize on the effect of initial macromolecular template on molecular modeling results.

2) Model 102D provided more hydrophobic participants when compared to 1D32. This might be expected since the residual constituents of 102D template included additional adenine and thymine bases with thymine being the most hydrophobic interacted residue.

3) All the compounds exhibited hydrophobic contacts with a number of DNA nucleobases in model 102D but there were some exceptions in model 1D32. To explain more, compounds 2, 5, 8, 10, 14 & 18 contributed to hydrophobic contact with DG, DC, DG, DG, DG & DG nucleobases, respectively. Such priority of guanine over cytosine in hydrophobic binding might be demonstrated through additional aromatic ring of guanine and hence providing weak interactions with DHP molecules.

4) Thymine was the only residue participating in key H-bonds with indeno [1, 2-b]quinoline-9,11-diones in model 102D. Our binding maps showed that this priority could be best explained by additional oxygen acceptors and also NH donors of thymine.

5) In compounds such as 1, nitroimidazole substituent provided additional hydrogen interactions with H-bond donor atoms of DNA bases (supplementary material 3). 

6) In non-aromatized structures, molecular alignment of compounds 2, 8 and 10 associated with key H-bonds to carbonyl oxygen of DG2 nucleobase (1D32). 

7) Unlike the model 1D32, in model 102D all the non-aromatized compounds participated in key H-bond interactions via DHP NH site. Compounds 6-10 made H-bonds with sugar ring oxygen of nucleotide while binding template for compounds 1-3 was through thymine carbonyl oxygen. 

8) *N*-phenyl substituted compounds (4, 5, 11 and 12**) **contributed to additional hydrophobic interactions with DC7 and DG8 nucleobases of DNA (1D32). Such hydrophobic contacts were observed within DA18, DT8, DT9 and DT19 residues in model 102D.

*Molecular dynamic simulations*

Dynamic stabilities of two indeno [1, 2-b] quinoline-9,11-dione/DNA complexes were evaluated using MD simulations. Considering probable binding mechanisms, i.e. intercalation (1D32) and minor groove binding (102D), the ligand with highest binding energy was selected for further simulations in each case. 

Due to the planar structure of Compound 16, it was expected that this compound could bind to the DNA structure through intercalating between DG and DC nucleobases. Moreover; presence of two methyl groups at position 7 of quinoline moiety opposed steric hindrance on compound 18 and made its interaction more favorable within DNA minor groove when compared to the intercalation between DNA nucleobases. 

After solvation of the DNA-ligand systems in a dodecahedron box with TIP3P water and adding enough number of Na^+^ ions to achieve electro-neutrality, 5000 minimization steps was performed using the steepest descent method. Soaking of DNA-ligand system was carried out by 100 ps constrained NVT followed by 100 ps NPT ensembles. Following system equilibration, 50 ns MD production was performed in each case without any constraint.

**Figure 5:**
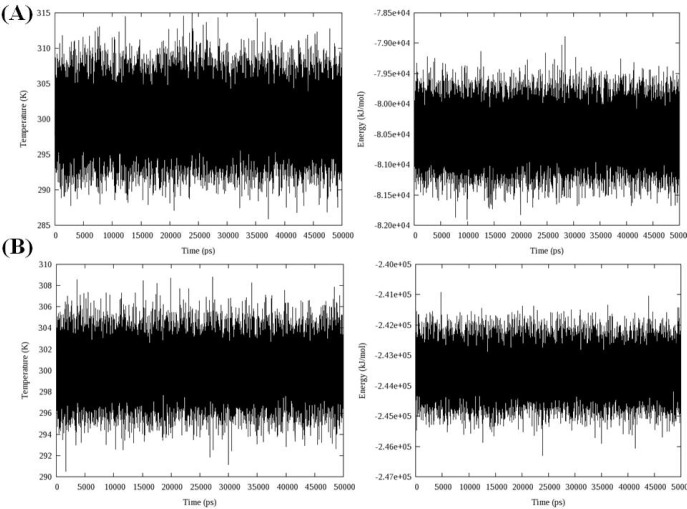
The time evolution of temperature and energy during 50 ns of MD simulations A: Complex of 16 and DNA (PDB code: 1D32), B: Complex of 18 and DNA (PDB code: 102D); data represents that conservation of energy over 50 ns MD simulations is convinced in both systems

Total energy, temperature and root mean square deviation (RMSD) were assessed to confirm the stability of trajectories. The average temperature during MD simulations at 300 K was found to be 299.0 K (± 3.6) and 298.2 K (± 2.3) in the case of models 1D32 and 102D, respectively. The temperatures of the both systems were stable for the whole simulation time. Fluctuation of temperature and energy is depicted in Figure 5. Obtained results showed that the conservation of energy over 50 ns MD simulations was convinced in both systems.

**Figure 6:**
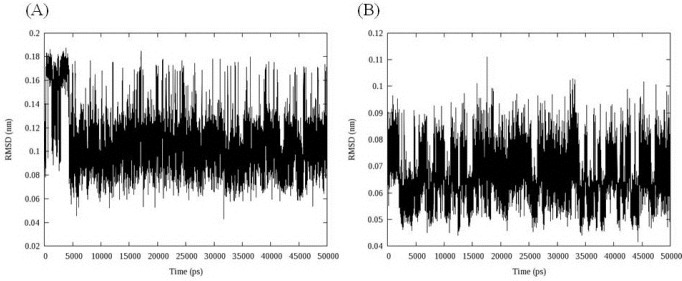
RMSD fluctuations of compound 16 (A) and 18 (B) in their complex with DNA over 50 ns MD simulations; data represented that both ligands were overally stable in their binding mode

The stability of the MD simulation was determined in terms of departures and fluctuations from the initial ligand-DNA structure. The time evolution of the ligand (all atoms) RMSDs was recorded as a function of time. The RMSD of two ligands with regard to initial conformations was illustrated in Figure 6.

**Figure 7:**
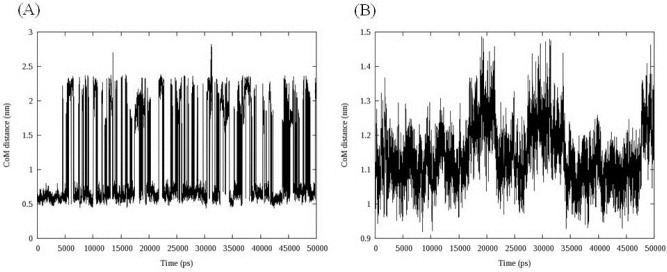
Center of mass (COM) distance between compound 16 (A), compound 18 (B) and DNA, data indicated that compound 16 showed dramatic changes in its COM distance during simulation time

As can be seen in Figure 7, both ligands had overall stability in binding state. Compound 16 bound to DNA through intercalation between DC and DG nucleobases. After 5 ns fluctuations, compound 16 converged to a stable binding conformation and leveled off the rest of MD simulations time. This demonstrated that after 5 ns simulation and initial fluctuations in the all atom scale of ligand RMSD, the ligand acquired an equilibrium state characterized by the RMSD outline. At first glance, the complex between compound 18 and DNA minor groove seemed to be unstable. But a closer look at RMSD fluctuations revealed that the changes in RMSD were very small by the mean 0.066 ± 0.008 nm.

More informative illustration of the system stability could be achieved by computing the center of mass (COM) distance between ligand and DNA. To get the DNA-ligand interactions, we calculated the COM distance between ligand and DNA during the simulation time. The results are depicted in Figure 7.

**Figure 8:**
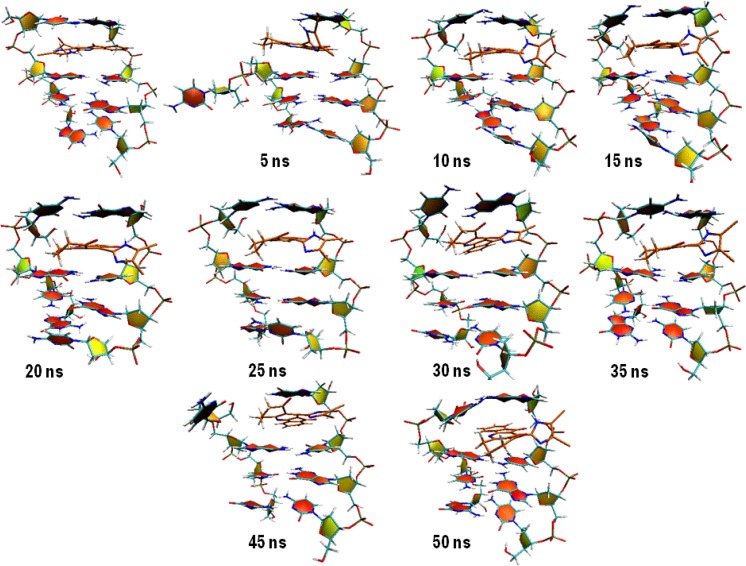
5ns snapshot representations of the complex between compound 16 and DNA (PDB deposited code: 1D32); as can be seen, the rise in COM distance might be related to the departure of DNA strands from initial position

Compound 16 showed dramatic changes in COM distance during simulation time. COM distance between the intercalated ligand (16) and DNA nucleobases was stable at mean distance 0.60 ± 0.05 till 4.54 ns when the first increase occurred. As can be seen in Figure 8, the new state seemed to be unstable and rapidly returned to the mean value. This trend repeated over the whole simulation time. To discover the system condition in these situations, complex structure between compound 16 and DNA was extracted from trajectories for each 5 ns (Figure 8). 

We postulated that compound 16 was probably capable of separating DNA strands from each other. The rise in COM distance might be related to the departure of DNA strands from initial position. This movement increased the COM distance between compound 16 and DNA with regard to their initial conformation (Figure 8A). The separation of DNA strands took place at the end of strand and we could not predict the situation in which the compound intercalated in the middle of DNA strands.

**Figure 9:**
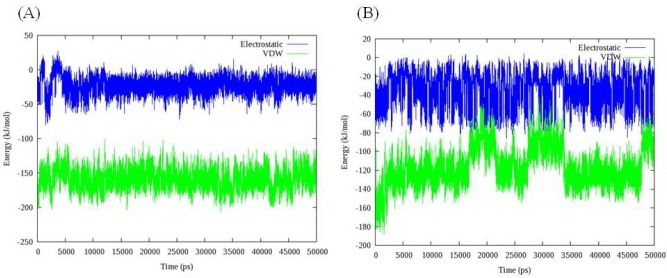
Electrostatic and Van der Waals interactions between compound 16 (A) compound 18 (B) and DNA

In the case of compound 18, three increases followed by relevant decreases in COM distance could be detected. The fluctuations occurred between 15 and 22 ns, 27 and 35 ns and finally at 47 ns and continued to the end of simulation. Double helix structure was stable during simulation so this time the movement of ligand could be responsible for observed changes. Compound 18 moved along the minor groove. In this case an interesting fact was the returning of ligand into its initial position. Analyzing the binding energy between compound 18 and DNA revealed that initial complex had maximum binding energy (Figure 9B).

**Figure 10:**
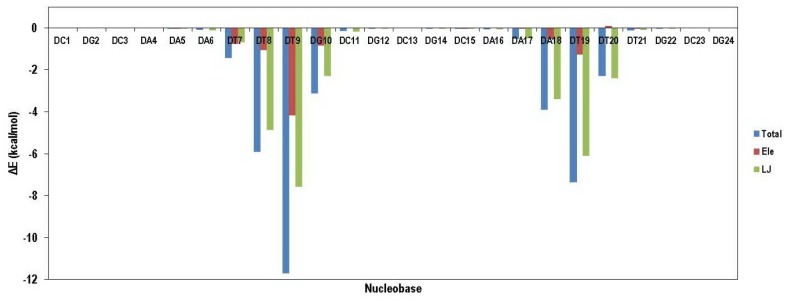
Contribution of various nucleobases in total binding energy of compound 18 in complex with DNA; data indicated that compound 18 preferred binding to DT reach domain in the minor groove of DNA mainly through VDW interactions while compound 18 could favorably bind to DT rich domain through free movement in the minor groove.

VDW interaction energy as a main contributor to total energy decreased due to the movement of ligand from its initial position. This result demonstrated that compound 18 preferred binding to DT reach domain. For more clarification, the contribution of each nucleobase in total binding energy is illustrated in Figure 10. As it was expected, compound 18 attached to the minor groove of DNA mainly through VDW interactions. Moreover; compound 18 could favorably bind to DT rich domain through free movement in the minor groove.

## Conclusion

The principal objective of the present study was to propose a binding model for cytotoxic indeno [1, 2-b] quinoline-9,11-diones as DNA interfering agents. Docking results indicated that these compounds could reversibly attach to DNA oligonucleotides with varied free binding energies ranging from about -6 to nearly 10 kcal.mol^-1^. Docking studies confirmed that the incorporation of electron-withdrawing substituents such as chlorine atoms to the imidazole moiety of indeno[1,2-b]quinoline-9,11-dions might be associated with higher cytotoxic effects MD simulation studies confirmed the stability of docked complexes and showed that probable binding mode of indeno [1, 2-b] quinoline-9,11-diones within DNA double helix could be intercalation between DC3, DC5, DG4 and DG6 nucleobases and indene, imidazole, indene and quinoline moieties of the ligands, respectively (PDB ID: 1D32). Linear regression analysis showed that cytotoxic effects of aromatized scaffolds in Jurkat cell lines may not be related to the DNA intercalation. MD simulations also demonstrated that compounds bearing two methyl substituents at their quinoline ring could attach to the minor groove of DNA by VDW interactions with DT rich domain through free movement in the minor groove. *N*-phenyl substituted indeno [1, 2-b]quinoline-9,11-dions contributed to additional hydrophobic interactions with DC7 and DG8 (PDB ID: 1D32) and DA18, DT8, DT9 and DT19 (102D) nucleobases of DNA. The applied method can be extended to other biological systems involving DNA-ligand interactions.
